# Biometric linkage of longitudinally collected electronic case report forms and confirmation of subject identity: an open framework for ODK and related tools

**DOI:** 10.3389/fdgth.2023.1072331

**Published:** 2023-08-04

**Authors:** Chrissy h. Roberts, Callum Stott, Marianne Shawe-Taylor, Zain Chaudhry, Sham Lal, Michael Marks

**Affiliations:** ^1^ Faculty of Infectious and Tropical Diseases, London School of Hygiene and Tropical Medicine, London, United Kingdom; ^2^ OPC Industries Ltd, Edinburgh, United Kingdom; ^3^ Hospital for Tropical Diseases, University College London Hospitals NHS Trust, London, United Kingdom; ^4^ Division of Infection and Immunity, University College London, London, United Kingdom

**Keywords:** fingerprints, biometrics, clinical trials, patient identification, electronic data collection, KoBoToolbox, ODK, open source

## Abstract

The availability of low-cost biometric hardware sensors and software makes it possible to rapidly, affordably and securely sample and store a unique and invariant biological signature (or biometric “template”) for the purposes of identification. This has applications in research and trials, particularly for purposes of consent, linkage of case reporting forms collected at different times, and in the confirmation of participant identity for purposes of safety monitoring and adherence to international data laws. More broadly, these methods are applicable to the needs of the billion people who live in resource-restricted settings without identification credentials. The use of mobile electronic data collection software has recently become commonplace in clinical trials, research and actions for public good. A raft of tools based on the open-source ODK project now provide diverse options for data management that work consistently in resource-restricted settings, but none have built-in functionality for capturing biometric templates. In this study, we report the development and validation of a novel open-source app and associated method for capturing and matching biometric fingerprint templates during data collection with the popular data platforms ODK, KoBoToolbox, SurveyCTO, Ona and CommCare. Using data from more than 1,000 fingers, we show that fingerprint templates can be used to link data records with high accuracy. The accuracy of this process increases through the linkage of multiple fingerprints to each data record. By focussing on publishing open-source code and documentation, and by using an affordable (<£50) and mass-produced model of fingerprint sensor, we are able to make this platform freely available to the large global user community that utilises ODK and related data collection systems.

## Introduction

The provision of official identification credentials is a crucial step in providing access to (among others) social and fiscal services, political franchise, free travel, birth registration and social engagement. An estimated one-billion people, and more than half of all people living in low- and middle-income countries (LMICs), possess no form of officially recognised identification credentials (1). Identifying, and later confirming the identity of individuals living as members of what The World Bank described as the “invisible billion” is a substantial challenge not only to excluded individuals and communities, but also to governments and other agencies acting in the interest of excluded communities. Within the sphere of public health work carried out in locales where official identification credentials are not available, there have been historical challenges when agencies have needed to identify, and to later confirm the identity of participants/subjects of research, clinical trials and interventions.

Clinical trials provide a powerful use-case for the development of novel systems for identifying and confirming identity of stakeholders from the invisible billion. Trials commonly aim to demonstrate the [a] efficacy and [b] safety of a public health intervention; outcomes that with rare exception require data collection to be longitudinal, heterogeneous, asynchronous and complex. The veracity of trial data is a paramount concern of Good Clinical Practice (GCP), the international standards for conduct and reporting of trials; but no gold standard method exists for confirming participant identities at different stages in the progress of a participant through a trial's protocol. This raises problems that [a] different case report forms (CRFs) which nominally relate to the same participant might in fact derive from two or more distinct individuals and [b] serious adverse events (SAEs) could be inappropriately attributed (or not) to the trial intervention. The first problem compromises the study design and conclusions, whilst the second could lead to the early halting (or inappropriate continuance) of the trial, false positive/negative results and serious patient, community or public health harms.

Such studies are bound by a range of national and international data laws such as the General Data Protection Regulation (EU, 2016/679). Among other laws of similar effect, GDPR (2) protects the data of participants (also known as “data subjects”) from improper processing, whilst simultaneously enshrining in law their broad-ranging legal rights to access, edit or request the erasure of data about them, including data derived from any information and biological specimens they may have provided during their participation. Thus, researchers must be able to effectively identify any participant and link them to their data in order to comply with requests for data deletion.

Digital authentication mechanisms based on biometric data have been proposed as a potential cornerstone in the future of inclusive and trusted identification methods (1). The recent availability of low-cost biometric hardware sensors and software makes it possible to rapidly, affordably and securely sample and store, alongside clinical trial (or other) data, a unique and invariant biological signature (or biometric “template”) of a study participant. By later being able to compare any two templates (i.e., from different CRFs), records can be linked and the identity can be confirmed in a way that is quantifiable, reproducible and automatable. Fingerprint templates are well described data entities that are regulated in the international standard ISO 19794-2:2005 (3, 4) and the interoperable ANSI INCITS 378-2004 standard. Templates do not take the form of a photograph or image of a recognisable fingerprint; rather they are an encoded text representation of the characteristic features of the fingerprint. Although iris (5), ear (6) and other sources of biometric templates have been explored and may be preferable in some contexts, fingerprints remain the most familiar form of biometric datum.

The development of novel biometrics systems has taken place alongside the rapid development of electronic data collection (EDC) systems. Historically, clinical trials relied on the use of paper CRFs, but over the last 15–20 years, there has been a widespread shift towards the use of electronic systems. The rate of adoption of EDCs in research and trials has been globally uneven, partly because of the relatively slower development of web infrastructure and limited penetration of the internet in some countries, regions and communities. A raft of EDC tools aimed specifically at work in resource-restricted settings have recently become available. These predominantly app-based tools, which include ODK (7), Ona Data (8), KoBoToolbox (9), SurveyCTO (10), CommCare by Dimagi (11) Enketo (12), REDCap (13), OpenClinica (14) and DHIS2 (15) all have the capability to function without reliable internet connections and can be used to generate digitised data sets of very high quality in even the most inhospitable and complex settings (16). Several of these platforms (ODK, SurveyCTO, KoBoToolbox, Ona and CommCare) are derived from a common code-base (ODK) and have overlapping functions, but none of these software platforms currently provide a robust built-in method for capturing verifiable biometric proof of identity data that can be used to later (a) link CRFs that were collected at different times or (b) confirm the identity of a participant requesting deletion of their data.

Low cost and portable biometrics sensors, used in combination with electronic data collection tools such as ODK, provide a potential solution to the problems of participant identification and CRF linkages. In this study, we report the development of a novel open-source app and associated method for capturing and matching ANSI INCITS 378-2004 fingerprint templates during data collection with ODK and related data tools. By focussing on the use of predominantly open-source code with an affordable (<£50) and mass-produced model of fingerprint sensor, we make this platform freely available to all global users of ODK, Ona, KoBoToolbox, SurveyCTO and CommCare.

## Materials and equipment

**Figure 1:**
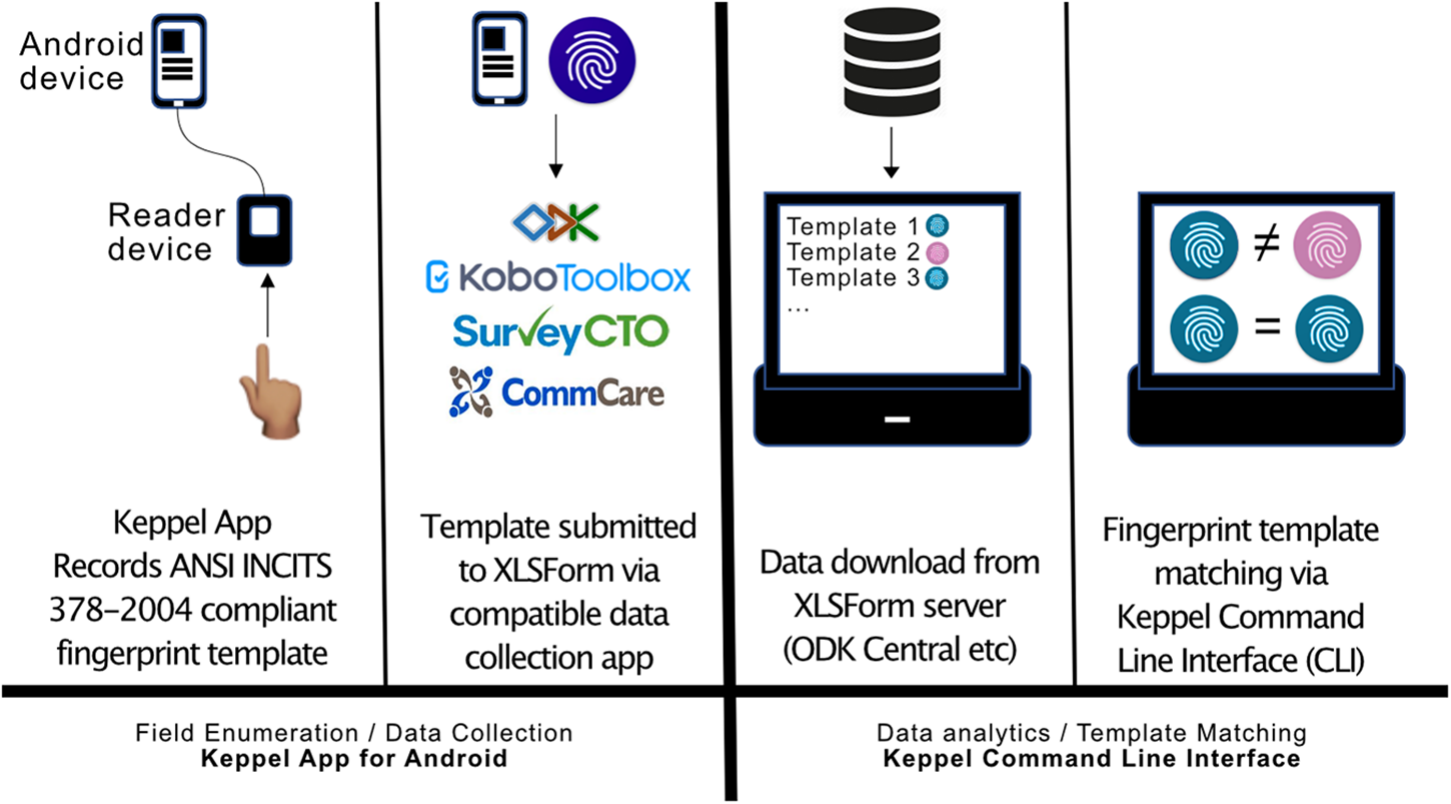
Schematic diagram of an open biometric framework for ODK and related tools. A portable fingerprint reader is connected to the enumerator's Android device. Via our Android app “Keppel”, which communicates directly with common electronic data collection apps (ODK Collect, Kobo Collect, CommCare, Survey CTO) an ANSI INCITS 378-2004 compliant fingerprint template is stored in an XLSForm and submitted to a web-server. After downloading all submissions of study forms, a separate command line interface (Keppel CLI) is used to carry out pairwise comparisons of templates, returning a matching score and match/mismatch result.

The novel biometrics system described here consists of two components (Figure 1). The first component is the “Keppel App for Android”, a smartphone app designed to run on Google Android operating systems. This app provides an input/output (I/O) interface between the ODK Collect app and an ANSI INCITS 378-2004 compliant electronic fingerprint reader/sensor device. The Keppel app for Android was designed and can be modified using Android Studio and Software Development Kit (SDK) (17). The initial version of the app works with the low-cost Mantra MFS100 Biometric C-Type Fingerprint Scanner (18), functionality for which was based on code templates provided within the Mantra MFS100 Software Development Kit (19). The second component of the system is the Keppel Command Line Interface (Keppel CLI), a Java application designed to run on the command line of a desktop, laptop or server-based workstation.

To fully implement this platform, users will need to have access to (1) a smartphone, tablet or laptop running a modern version of Android (2), a compatible data collection tool [ODK Collect, SurveyCTO Collect, CommCare, KoboCollect] (3), copies of the Keppel app and CLI (4), a Mantra MFS100 Biometric C-Type Fingerprint Scanner and (5) a personal computer capable of running a UNIX-like operating system (including Linux, UNIX and Windows Subsystem for Linux).

## Methods

### Development of the ODK biometrics framework

The Keppel app acts as an intermediate between the hardware scanner and the client data collection software app (i.e., ODK Collect) and is configured from within the form designer on the client side. Scanning a fingerprint using the app captures and then copies a plain text representation of a fingerprint template to the client data collection app. The Keppel app was designed with a view to making the addition of further biometric sensors relatively simple through the implementation of a common scanner interface in the code-base. A software “demo” scanner, which returns simulated fingerprint data, is also included. This allows users to test their fingerprint supported ODK forms without having a scanner connected.

The Keppel CLI application is able to compare any two ANSI INCITS 378-2004 fingerprint templates and to generate a simple unitless score (S) which describes the overall similarity between the two templates. The Keppel Java CLI utilises an existing open-source library (20) to calculate the similarity between fingerprint templates. Calls to the CLI take the form “keppel match -p [template1] [template2]” where [template 1] and [template 2] are plain text copies of the fingerprint templates of interest. The CLI returns either the absolute value of S (i.e., 193), a logical test of whether S is greater than a provided threshold (T) (i.e., matched/mismatched), or both (i.e., matched_193). Details of the underlying algorithm used for template matching are provided in a fully transparent way by its originator (20). We also reproduce a copy of relevant information in the Supplementary Information.

Full details on how to configure and use the Keppel app, along with example images of the user experience are provided in the Supplementary Material and in the code repository for the project, which is available on GitHub (21).

### Compatibility

We tested the Keppel app with current releases of data collection apps including ODK Collect (v2022.3 Beta 0), SurveyCTO Collect (v2.72), CommCare (v8) and KoboCollect (v2022.1.2). The Keppel CLI was tested on Ubuntu (Linux) v18.04.4, Darwin (Unix) v21.5.0 and Windows Subsystem for Linux (WSL) 2.

### Data collection

Data were collected on encrypted Android handsets using ODK Collect (16) and were hosted at institutional data centres. We recruited volunteers to provide fingerprints for a real-world evaluation of the system. Participants provided their age, gender, skin-tone (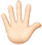 light |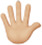 medium-light | 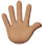 medium | 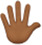 medium-dark | 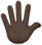 dark), natural level of skin moisture (very dry | dry | normal | moist | very moist), skin greasiness (not greasy | mildly greasy | greasy | very greasy), and overall condition of skin (very bad | bad | good | very good).

Participants were then asked to sequentially scan each of the fingerprints of their right hand on the fingerprint reader. They then repeated this process, recording the same fingerprint a second time to create a matched pair of templates for each of their five fingers. For each of the five fingers, participants indicated whether that finger had visible rough patches (yes/no) and/or smoothed areas (yes/no). This set of matched template pairs represented the true positive group in the analysis. To establish a true negative group, the first five fingerprint templates of each individual were paired with the first five fingerprint templates of another individual from the study. In each case, we ensured that the thumb, index, middle, ring and pinkie prints from the right hand of individual A were paired to the same fingers of individual B's right hand.

### Analysis

We aimed to recruit a minimum of 200 individuals, generating more than 1,000 fingerprints to include in our initial evaluation. Linear regression was used to identify skin characteristics that were associated with changes in the average score among the true positive template pairs. The Receiver Operator Characteristic (ROC) was used to assess the overall performance of the matching algorithm and to identify suitable threshold values for determining matched and unmatched classifications. We used the area under the ROC curve (AUC) as a generalised indicator of the matching algorithm's performance. The AUC value is an indicator of the aggregate performance of a binary diagnostic assay across all possible classification thresholds. AUC = 1 indicates a perfect classifier and AUC = 0.5 indicates a classifier that performs no better than a random coin-toss. AUC provides a simple way to compare the performance of two diagnostics in a way that is independent of scale and threshold values, and when comparing several classifiers, the one with the higher AUC is generally the more performant assay. In order to determine whether the accuracy of matching could be increased by using more than one finger in the classifier, we calculated a multi-finger score (∑S) by summing the values of S from two (thumb, index), three (thumb, index, middle), four (thumb, index, middle, ring) or five (thumb, index, middle, ring, pinkie) fingers. ROC analysis was performed and the optimal assay was chosen on the basis of maximising the AUC whilst minimising the number of fingers used to perform the test. All analysis was performed in R v4.2.0.

## Results

All code and working software releases of the app and CLI (v0.3) are provided in Supplementary File S1. The most recent version of the app and CLI are available from our code repository (21).

### Template matching

**Figure 2:**
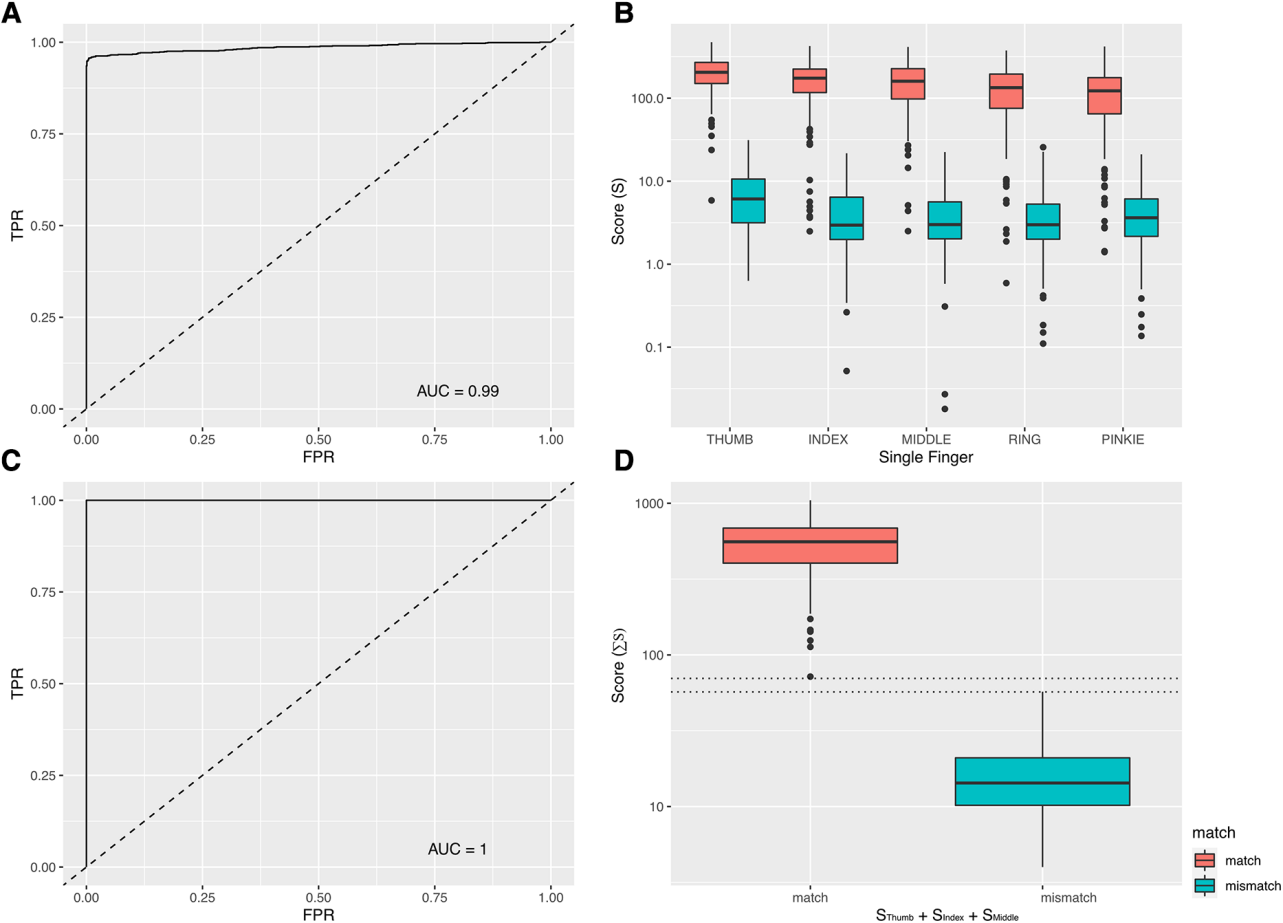
(**A**). ROC analysis (Single Finger). The area under the ROC curve for any pair of fingerprint templates was 0.99. This analysis was based on 1,010 true positive and 1,010 true negative template pairs. (**B**) Assay performance variations by finger. There was a progressive (from thumb to pinkie) decrease in the average score [S] of a true positive template pair according to which finger was used. (**C**) ROC analysis (score summed across thumb, index and middle finger). The area under the ROC curve was 1.00, indicating a perfect delineation between true positives and true negatives. (**D**) Summed scores [∑S] of three-finger scanning among true positive and true negative groups.

Overall, 1,010 true positive fingerprint template pairs (*n* = 2,020 individual templates) were provided by 202 consenting participants with different skin tones, qualities and conditions (Table 1). 1,010 true negative pairs were synthesised as described above. The mean score for a comparison between templates of a true positive pair was *S* = 163.0 (median 159.4, min 0.6, max 472.7, IQR 97.0–219.5). For true negative template pairs, the mean score was *S* = 5 (median 3.6, min 0.0, max 31.3). ROC analysis of 1,010 true positive and 1,010 true negative template pairs revealed an area under the curve (AUC) of 0.99 (Figure 2A). At the threshold score (T) of *T* = 27, the fingerprint matching algorithm had a false positive rate of 0.001 and true positive rate of 0.95. A threshold of *T* = 27 was subsequently used as the cut-point between mismatched (*S* < 27) and matched (*S* ≥ 27) template pairs. A total of 52 (5.14%) true positive pairs were falsely classified as negative. Among the true negative template pairs, just one pair returned a false positive result (*S* = 31.26).

**Table 1:** Characteristics of study participants and their skin.

Group	Subgroup	*n*	%
Age Group	20–30	104	51.5
	31–50	85	42.1
	51+	13	6.4
Skin Tone	Dark	3	1.5
	Medium dark	8	4.0
	Medium	32	15.8
	Medium light	59	29.2
	Light	100	49.5
Skin Moisture	Very dry	6	3.0
	Dry	72	35.6
	Normal	110	54.5
	Moist	14	6.9
Skin greasiness	Not greasy	105	52.0
	Mildly greasy	85	42.1
	Greasy	12	5.9
Skin Condition	Bad	21	10.4
	Good	157	77.7
	Very good	24	11.9
Creams or lotions used today	No	109	54.0
	Yes	93	46.0
Oils or emollients used today	No	183	90.6
	Yes	19	9.4
Medication used today	No	195	96.5
	Yes	7	3.5
Hand sanitiser used today	No	48	23.8
	Yes	154	76.2
Surgical scrub used today	No	106	52.5
	Yes	96	47.5

The majority of participants (*n* = 170, 84.16%) received positive matches (*S* > 27) for all five fingers. Of the remaining 32 participants, 18 (8.91%) received positive matches for four fingers, with nine (4.46%), two (0.4%) and one (0.1%) participants receiving positive matches at three, two or one fingers respectively. Compared to the average values for thumb templates, relative values of S (RS) were significantly and progressively lower for template pairs representing the index (RS = −34.2, SE = 8.0), middle (RS = −43.7, SE = 8.1), ring (RS = −68.6, SE = 8.1) and pinkie (RS = −87.0, SE = 8.1) fingers (Figure 2B and Table 2).

**Table 2:** Linear regression analysis: Factors associated with increased or decreased scores.

Factor	RS*	SE**	*P*
(Intercept)	228.19	23.96	<0.001
Thumb (Ref)	REF	REF	REF
Index Finger	−34.24	8.04	<0.001
Middle Finger	−43.71	8.06	<0.001
Ring Finger	−68.64	8.05	<0.001
Pinkie Finger	−86.95	8.06	<0.001
Rough Areas	−16.89	12.06	0.162
Smoothed Areas	1.20	7.17	0.867
Age 20–30	REF	REF	REF
Age 31–50	9.28	5.56	0.095
Age 51+	−70.86	11.85	<0.001
Skin Tone: Dark	REF	REF	REF
Skin Tone: Medium Dark	−11.46	26.45	0.665
Skin Tone: Medium	−23.40	23.53	0.320
Skin Tone: Medium Light	−16.52	22.88	0.470
Skin Tone: Light	−14.62	22.69	0.520
Skin Moisture: Normal	REF	REF	REF-
Skin Moisture: Moist	1.03	11.02	0.925
Skin Moisture: Dry	−0.79	5.90	0.893
Skin Moisture: Very Dry	17.24	16.37	0.292
Skin: Not Greasy	REF	REF	REF
Skin: Mildly Greasy	5.97	5.65	0.290
Skin: Greasy	−0.39	11.74	0.974
Skin Condition: Good	REF	REF	REF
Skin Condition: Bad	−6.63	9.00	0.462
Skin Condition: Very Good	26.43	8.62	0.002
Applied Lotion/Cream Today	−11.59	5.86	0.048
Applied Emollient/Oils Today	−10.21	10.04	0.309
Applied Medication Today	3.05	16.80	0.856

* RS: The average relative value of the matching score S, compared to the reference group (REF) for factors with multiple responses, or compared to the counterfactual for factors with binary responses.

** SE, standard error.

Average relative scores for true positive template pairs were lower when collected from people aged over 50 (RS = −70.9, SE = 11.9) and higher when collected from people who described their overall skin condition as “very good” (RS = 26.4, SE = 8.6). There appeared to be no variation in the average score of a true positive template pair on the basis of skin tone, moisture level, or greasiness (Table 2). There was some evidence for a slight reduction in scores when the participant had applied creams or lotions to their hands on the day of sampling (RS = −11.6, SE = 5.9) although this did not appear to be confirmed among those who had applied oils, emollients or medications. The presence of rough or smooth patches on the fingertips did not appear to affect the average score (Table 2).

There was a 94.9% probability that any two fingerprint templates taken from a single finger would return a positive match (defined as *S* > 27). The probability that there would be at least one positive result among any two template pairs taken from two fingers of the same individual was 99.0%. Using three, four or five fingers, the probability of returning at least one positive result (*S* > 27) rose to 99.6%, 99.8% and 100% respectively.

Using the sum of the scores from two (AUC = 0.997), three (AUC = 1.0), four (AUC = 1.0) or five (AUC = 1.0) fingers indicated a general improvement in the performance of the matching algorithm with an increasing number of fingers used. In this data set, we found that a perfect classifier (AUC = 1) could be achieved when three fingers (thumb + index + middle) were used (Figures 2C,D). In the three-finger model, the mean summed score for a true positive was ∑S = 557.18 (median 557.18, min 71.94, max 1,046.43, IQR: 402.79–686.03). For true negatives, the mean summed score was ∑S = 16.53 (median 14.3, min 3.99, max 57.22) (Figure 2D). The gaps between the highest values of a true negative and the lowest values of a true positive in each classifier were 13 (three fingers), 12.3 (four fingers) and 62.7 (five fingers) units. This indicated that among the three classifiers where AUC = 1.0 (i.e., the models where three, four or five fingers were combined), there was an additional performance gain from using more fingers, as the separation between the positive and negative groups (and consequently the proportion of all possible threshold values where the classifier worked perfectly) increased.

### System compatibility and performance

The CLI was highly performant. Using a single CPU on a 2019 MacBook Pro (2.3 GHz, 8 Core Intel i9, 64 GB RAM), we compared 400 pairs of templates in 140 s, which equated to 0.350 s per comparison. Using a 16-core parallel implementation through the R package “furrr” (22), we processed 2,000 template pairs in 69.7 s, equating to 0.035 s wall-clock time per comparison. The Keppel CLI worked as expected on Linux, Unix and within the Windows Subsystem for Linux. We tested compatibility of the Keppel app with several commonly used data collection apps that are based on ODK's code. We found that our biometrics app functioned as expected with ODK Collect (v2022.3 Beta 0), SurveyCTO Collect (v2.72) and KoboCollect (v2022.1.2). Although we anticipate that the system can work with CommCare, we were not able to confirm compatibility with a current version of the CommCare app (v8) because required functions (external app integration) were paywalled in CommCare, only being made available for higher tier subscribers of that service. Ona does not have a standalone data collection app and users instead connect to Ona servers via ODK Collect, therefore making it compatible with the Keppel app. The system is currently not compatible with Go.Data, DHIS2 or OpenClinica.

## Discussion

Scant literature addresses topics of participant (mis)identification in research and trials, but in clinical care the subject is better explored (23, 24) and highlights that patient mis-identification is common even in high income countries (HICs) with well-developed health information systems. In resource-restricted settings, challenges are magnified, particularly when complex longitudinal data collection centres around the use of experimental medical products, or the surveillance of infectious diseases in remote locales (16).

The current generation of mobile data collection software is well developed for use in these settings, with off-grid functionality the norm; but no system historically provided a simple to use method to support participant identification. We have created open-source code and related applications that introduce options for fingerprint template capture, and workstation-based template matching when collecting data with ODK's native smartphone app ODK Collect. We have tested and confirmed that the app also works with the majority of other software that have been forked (copied and further developed as separate software, usually by an independent developer group) from the ODK project. This includes KoBoToolbox, Ona and SurveyCTO, which together with CommCare (compatibility unconfirmed) and ODK have a dominant market share of mobile data collection tools used in public health, humanitarian activities, election monitoring and elsewhere. The system is not currently compatible with major data collection systems that are not based on ODK's code (i.e., OpenClinica, DHIS2, REDCap and Go.Data).

There appeared to be several factors that significantly influenced the quality of template matching. Most importantly, we highlighted that for the purposes of clinical trials, it was insufficient to rely on scanning just one fingerprint template in each CRF. The data presented here indicate that the matching of a single fingerprint template pair has absolute accuracy of around 95%. This is perhaps acceptable for confirming identity during the process of a data subject access request, where any issues of false-negative results might be rectified in most cases by trying again with a new scan from the same finger. 95% accuracy is meanwhile too low for the practical purposes of needing to confirm links between data. A recent clinical study protocol aimed to vaccinate 500,000 participants (25). Assuming 10 CRFs per participant and single scanning of fingerprint templates on each CRF, then we estimate that there would be 5,000,000 comparisons, of which around 250,000 linked records would not match because of the 5% error rate. At such scales, subtle (i.e., 3–4 decimal places) increases in classifier performance could translate into substantial decreases in the actual number of errors in a real world setting. By using multi-finger scanning (and then summing the matching scores), we reduced the error to levels that were undetectable in our test data. We also saw that whilst our relatively small data set was underpowered to discern the subtle performance increases in AUC of a ROC analysis when four or five fingers were used (compared to three fingers where AUC = 1), there was evidence from the growing separation of the negative and positive populations that using more fingers would continue to decrease error in the system and improve accuracy. We therefore recommend that all users of the system should collect a minimum of three and ideally more fingerprint templates in each CRF. We found that the average score for a matched pair was highest for thumb templates and that each subsequent finger moving towards the pinkie had a lower average score. When choosing which fingers to scan, it may be valuable to prioritise fingers accordingly.

Certain participant characteristics influenced the probability of obtaining a high matching score from a pair of templates collected from the same finger. We found that template pairs from those who self-described as having “very good” skin condition were more likely to produce high scores. The score neither appeared to be influenced by skin tone or greasiness, nor by the presence of rough or smooth areas. Template pairs from people aged over 50 were substantially lower (RS = −70) than those from people aged 20–30. In this study, we focused on the use of fingerprint templates taken from the right hand and we are therefore unable to determine whether there could be performance differences when using templates from the left hand.

The Keppel app and CLI is not the first biometrics platform that is compatible with the ODK ecosystem. Simprints (26) is well-developed software which is compatible with ODK-like systems, as well as with DHIS2. This platform openly shares code (27) but its use may require a managed account provided by the not-for-profit Simprints Technology Ltd. Use of Simprints also appears to require access to proprietary scanner hardware, access to which may also require direct collaboration with the company. Whilst Simprints does have wider functionality than the system presented here (including contactless biometrics and on-device matching/verification), our project is novel in being designed around low cost, off-the-shelf hardware and a fully “open software - open documentation” model which makes setup simple, which can be used by any project using compatible software and which does not require support from, partnership with, or subscriptions provided by any third party.

Most trials have tens, hundreds or thousands of participants, meaning that compute times for the Keppel CLI are negligible in most cases, even when multi-finger scanning and multiple CRFs are used. The CLI was however highly performant at scale and we predict that in a very large trial with 15,000,000 templates (500,000 participants, 10 CRFs, three-finger scanning) the CLI could process all required template comparisons in around one week of wall-clock time on a higher-end consumer laptop (2.3 GHz, 8 Core Intel i9, 64 GB RAM). Using a high-performance cluster would reduce this in proportion to the number of CPUs available (for instance, wall clock time would be around 15 min if using a 96 CPU cluster).

Limitations of this study include that the first release of the Keppel app does not perform on-device matching or verification, and that the app works with just one specific and proprietary hardware scanner device. To ameliorate the risk that the compatible hardware may become obsolete, we encoded the app in such a way that further devices (including sensors for other biometrics) could be added with relative ease. Our open-source code is also available for community-led maintenance and further development. This study was performed during the COVID-19 pandemic at a time when collecting fingerprints was a non-trivial process. As such, we did not achieve a sample size that would have allowed us to estimate with greater precision the level of error that remained when performing four- or five-finger scanning. Our error estimates are therefore indicative and should be interpreted only to show that multi-finger scanning is the preferable method of sampling. We were also unable to include children and infants in the study (because of risk assessments related to COVID-19) and are consequently not able to use these data to assess whether the system performs well in young and very young people. This will be a topic of future study.

The use-case for these tools in clinical trials, medical informatics, research and health care is extensible to the broader use-cases of humanitarian electronic data management tools. Central to the value of low cost and reliable bioinformatics tools in action for public good are deployments that will address the problems of the excluded “missing billion”. We anticipate that the tools presented here, along with those which will emerge from continuous open-source developments of the code-base, will contribute to efforts that will provide official and semi-official identification credentials; thereby enhancing political franchise, supporting free and fair elections, enabling birth registration & census and providing solutions to many other personal identification challenges in resource-restricted settings.

## Data Availability

The datasets presented in this article are not readily available because fingerprint data cannot be shared for ethical reasons. All new code and software required to use the platforms described in the paper are provided in the Supplementary Files. The latest release of the app and CLI, along with all code relating to this work, are available at the project home page: https://github.com/LSHTM-ORK/ODK_Biometrics. Copies of all code and release version 0.3 are provided as Supplementary Data. The Keppel App runs on Android Devices. The app works in combination with one of ODK Collect, KoBo Collect and SurveyCTO Collect but may work with other similar apps. The Keppel CLI was programmed in Kotlin and is platform independent. All code is released on the MIT Licence (https://opensource.org/Licenses/MIT). At the time of writing, the Mantra MFS100 Biometric C-Type Fingerprint Scanner was widely available from online retailers as well as from the manufacturer www.mantratec.com. Some code used in this project was based on the Mantra MFS100 Software Development Kit, which at time of writing was available from Mantra Softech https://download.mantratecapp.com/. Requests to access the datasets should be directed to chrissy.roberts@lshtm.ac.uk.
