# Supplementary material for: Biometric linkage of longitudinally collected electronic case report forms and confirmation of subject identity: an open framework for ODK and related tools
Source: Front Digit Health. 2023 Aug 4;5:1072331. doi: 10.3389/fdgth.2023.1072331 (PMC10436742; doi:10.3389/fdgth.2023.1072331)
Supplement: Supplementary file 3 [file Datasheet3.pdf]

Keppel

Android failing

CLI passing

This project provides a simple Android app that interfaces with mobile data collection software to allow fingerprint templates to be scanned as part of an XLSForm. We also provide a second app, designed to be run on a computer workstation, which can compare two fingerprint templates and return a matching score.

Compatibility

This platform should work with all platforms that are based on ODK.

Platform	App & version	Compatibility
ODK	ODK Collect v2022.3	YES
KoBoToolbox	KoboCollect v2022.1.2	YES
SurveyCTO	SurveyCTO Collect v2.72	YES
CommCare	CommCare v8	Only with Advanced Plan or higher
Ona	ODK COllect v2022.3	YES

System design

The novel biometrics system consists of two components. The first component is “Keppel”, a smartphone app designed to run on Google Android operating systems. This app provides an I/O interface between the ODK Collect app and an ANSI INCITS 378-2004 compliant electronic fingerprint reader/sensor device. The app has to be sideloaded (it isn't on play store yet).

A really important point here is that the system is not simply taking photographs of fingerprints. The data are stored as concise code which has a very 'lite' impact on the size of the data stored in ODK and also requires no use of attachments. The fingerprint data are captured as plain text that is stored and encrypted along with other ODK data.

The Keppel Smartphone app was designed using Android Studio and Software Development Kit (SDK). The initial version of the app works only with the low cost (<£50) Mantra MFS100 Biometric C-Type Fingerprint

Scanner from [Mantra Softech Inc](), functionality for which was based on code templates provided within the [Mantra MFS100 Software Development Kit]().

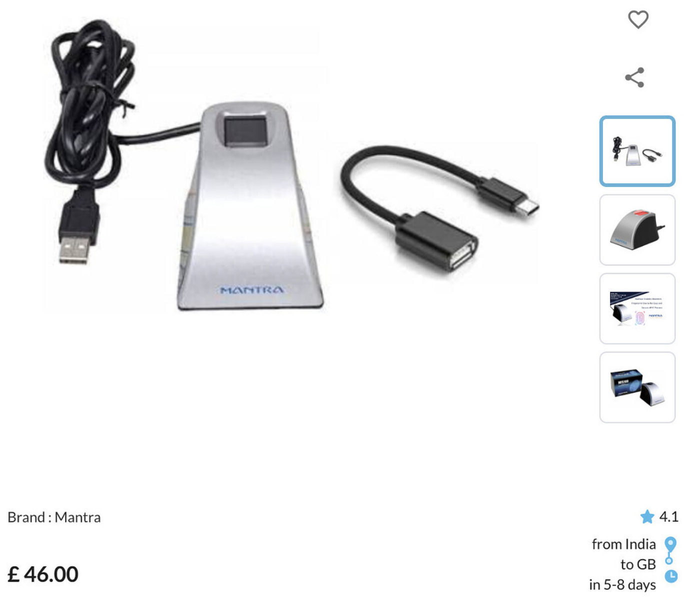

The app was designed with a view to making the addition of further biometric sensors relatively simple. A software ‘demo’ scanner is also included, and this allows users to test their fingerprint supported ODK forms without having a scanner connected.

Security and Privacy

Please be aware that fingerprints (or any form of biometric data) are very sensitive personal data. Collecting them may help your programme or study but make sure to fully consider privacy and security concerns when doing so. Please consider carrying out a data protection impact assessment prior to data collection.

If you're collecting any personal information using ODK it would be a good idea to look into [encrypting forms]() and also to read ODK's [general documentation on security]().

Usage

Install the Keppel App on your Android phone or tablet

Download the [latest release]() and sideload the APK file on to your Android device

Scanning fingerprints in ODK XLSform format

To setup a form to scan fingerprints the devices used for data collection will all need the app installed. It can be downloaded [here](). The app integrates with ODK Collect's [External app widget]() using the `uk.ac.lshtm.keppel.android.SCAN` intent. An example [XML form]() and [XLS Form]() are provided.

Forms should be added to your ODK system as usual, via ODK Central.

To capture all the fingers of one hand, your XLS form would look like this.

	type	name	label	appearance	required	bind::odk:length	constraint
1	type	name	label	appearance	required	bind::odk:length	constraint
[illegible]	[illegible]	[illegible]	[illegible]				
34	begin_group	scan1.	**Please Scan the fingers of the right hand**	**field-list**			
35	text	R.THUMB	Right Thumb	ex:uk.ac.lshtm.keppel.android.SCAN		10000	
36	text	R.INDEX	Right Index	ex:uk.ac.lshtm.keppel.android.SCAN		10000	
37	text	R.MIDDLE	Right Middle	ex:uk.ac.lshtm.keppel.android.SCAN		10000	
38	text	R.RING	Right Ring	ex:uk.ac.lshtm.keppel.android.SCAN		10000	
39	text	R.PINKIE	Right Pinkie	ex:uk.ac.lshtm.keppel.android.SCAN		10000	
40	end_group						

The images below show how this looks in ODK Collect. Clicking 'launch' in ODK Collect opens the external app. Pressing 'capture' activates the scanner. Once the template has been captured, the data are returned to ODK Collect as plain text (N.B. Here I'm using the dummy scanner)

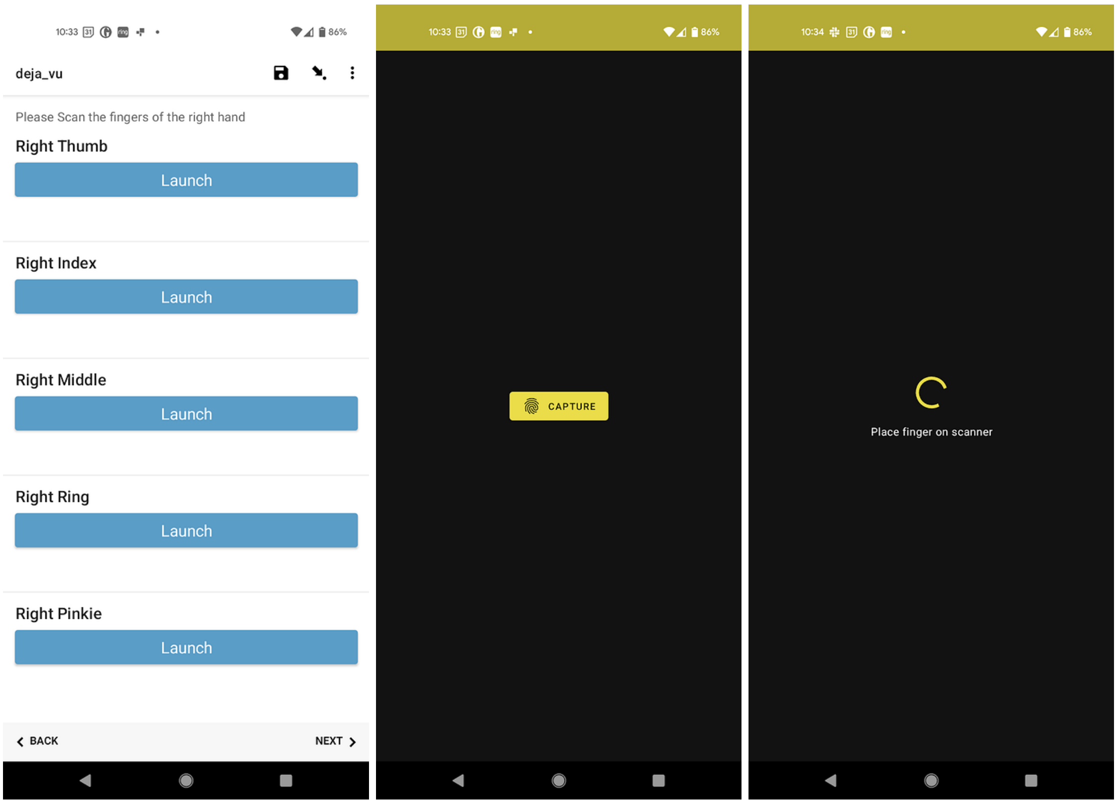

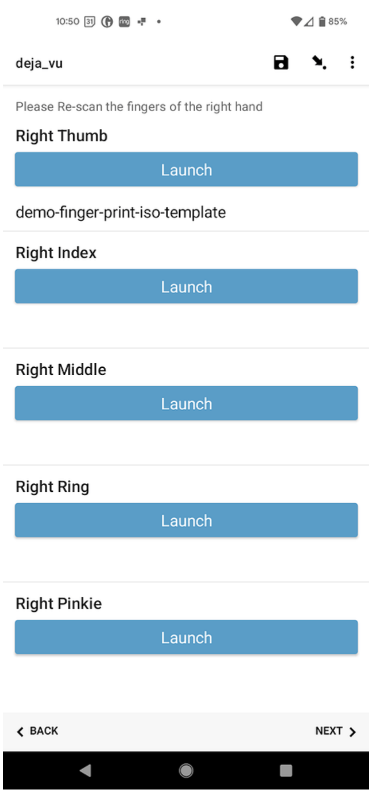

ODK data downloads

When you download your data CSV file from ODK Central, your fingerprint templates will be stored as plain text in line with other data from the form. From here you can either test them one at a time, or use a script to automate batch processing.

SubmissionDate	ID	scan1.-R.THUMB	UUID
2021-12-21T18:19:36.622Z	001	464d5200203230000000000f60000013c016200c500c5010000006424808b00ca7664...	uuid:4f1f19e2-846a-42d7-aef2-0574474a993b
2021-12-21T19:25:32.123Z	002	464d52002032300000000009c0000013c016200c500c5010000003415408c00e0874c...	uuid:2382975e-52bb-4a6b-880f-49bc5c27e787
2021-12-22T09:14:09.431Z	003	464d5200203230000000000ae0000013c016200c500c5010000006418409200cb7b...	uuid:f7a0ab80-9d46-423a-9e64-28a0b3fb7076
2021-12-22T12:00:11.682Z	004	464d5200203230000000000d80000013c016200c500c501000000461f807f00eed71...	uuid:9d55c0a9-901a-4908-b553-8a43a0b4037e

Install the Keppel CLI on your workstation

Unzip the keppel-cli.zip. Open a terminal and do the following to copy all required applications and libraries to your /usr/local/bin folder

```
foo@bar:~$ cd keppel-cli
foo@bar:~$ ./install.sh
```

Test the installation with

```
foo@bar:~$ keppel
```

you should see the help dialog

```
foo@bar:~$ keppel

Usage: keppel [OPTIONS] COMMAND [ARGS]...

Options:
  -h, --help  Show this message and exit

Commands:
  match  Match two hex encoded ISO fingerprint templates. Threshold used for
         matching is 40.0.
```

Matching fingerprints

To match two (hex encoded) fingerprint templates run:

```
foo@bar:~$ keppel match /path/to/first_template /path/to/second_template

15.386568130470566
```

The core function requires that each template is stored in a single line of its own text file. The default behaviour is to return the matching score for the two templates

Other commands

To see available commands type

```
foo@bar:~$ keppel match -h
```

From version 0.3, the following options are available

-p
Treats TEMPLATE*ONE and TEMPLATE*TWO as plain text rather than file This option is very useful for scripted analysis

Example [templates truncated]

```
foo@bar:~$ keppel match -p 464d520020323000000001080000013c016200c500c5... 464d520020323(

15.386568130470566
```

-ms
Return whether templates match along with score like "match_210.124"

-m
Return whether templates match (either "match" or "mismatch")

-t FLOAT
Threshold (score) to be used to determine whether templates are a match or mismatch

-h, --help Show this message and exit`

Controlling the Keppel CLI with R

R is our favourite application for data munging, analysis and statistics. R plays nicely with system tools and can be used to control the Keppel API. .

In R, we created a very simple wrapper function that runs the Keppel CLI and returns a match score. As with any R function, this can be applied to lists, arrays and tibbles (data frames) to perform batch actions.

```
##########################################################################
#Define a function that gets the match score between scans.
##########################################################################

fingerprint.score<-function(print1,print2)
{

  if(!is.na(print1) & !is.na(print2)){
    c<-system(
      command = str_c("keppel match -p ",print1," ",print2),
      intern = TRUE
    )}

  if(is.na(print1) | is.na(print2)){ c<-NA}
  message(c)
  c
}
```

On an off-the-shelf MacBook Pro with 2.3 GHz 8-Core Intel Core i9 and 32 GB RAM, it took approximately 71 seconds to process 200 template matching calls sequentially.

```
library(furrr)
library(future)

plan(sequential)

system.time(future_map2(
  .x = df$scan1,
  .y = df$scan2,
  .f = fingerprint.score))

user     system  elapsed
101.276  16.056  71.427
```

Called in parallel using the ***furrr*** and ***future*** packages, the process is much faster

```
library(furrr)
library(future)

plan(multisession, workers = 16)
system.time(future_map2(.x = df$scan1..R.THUMB,.y = df$scan2..R.THUMB.2,.f = fingerprint.

user   system  elapsed
1.261  0.059   16.467
```

Processing n templates using the parallel approach (16 cores) took

n	Cores	time (s)	time/call (s)
200	1	68.6	0.343
400	1	140.2	0.350
200	16	16.5	0.083
400	16	30.86	0.077
1000	16	25.0	0.025
2000	16	69.7	0.034
10000	16	773.4	0.077

Creating an Android release

Hopefully the current release will continue to work with future versions of ODK Collect. At present we are passively updating the app as needed. Collaborators are welcome to continue to develop the app and to create new releases.

Prerequisites: You will need to install both a JDK and the Android SDK to build a release. The easiest way to install Android is to download [Android Studio] and import the project in `Android` into it. This takes care of downloading the correct Android dependencies for you.

To create a release:

1. Update the `versionCode` and `versionName` in `Android/app/build.gradle` for the release. `versionCode` should be any number higher than the current value.
2. Commit the changes: `bash git add Android/app/build.gradle git commit -m "Update versionName and versionCode"`
3. Tag the latest commit and push the changes: `bash git tag <versionName> git push git push --tags`
4. Run `./import-device-sdks.sh` and follow any instructions to make sure device SDKs are setup correctly
5. Run `./build-android-release.sh` to build the signed release APK
6. Navigate to https://github.com/chrissyhroberts/ODK*Fingerprints*Mantra/tags, click your new tag, click "Edit tag"
7. Set the `versionName` as the "Release title", attach the signed release APK and hit "Publish release" 🚢

Creating a CLI release

Prerequisites: You will need to install a JDK

To create a release:

1. Tag the latest commit and push: `bash git tag <versionName> git push --tags`
2. Run `./build-cli-release.sh` to package the CLI
3. Navigate to https://github.com/chrissyhroberts/ODK*Fingerprints*Mantra/tags, click your new tag, click "Edit

tag"
4. Set the "Release title", attach the `.zip` package and hit "Publish release" 🚢

Supported devices

Mantra MFS100

Mantra Softech India Private Limited

- Mantra is a leading manufacturer and developer of Biometric devices, software and solutions from 2006 onwards.
- The biometric-based technology offers a dependable, helpful, and authentic way of verifying/identifying an individual's identity utilizing latest Biometric fingerprint scanner.
- Fingerprint scanners are being used broadly for enrollment, identification & verification in varied projects where the identity of people is required.
- Mantra's biometric fingerprint devices offer superior execution, accuracy, and continuance.
- Our wide ranges of fingerprint scanner devices deliver quick personality verification with a high level of security in a consistent way for various Citizen or Person identity ventures.

Mantra’s MFS100 - Biometric Fingerprint Scanner

- FBI and STQC certified single finger scanner MFS100 is high quality USB fingerprint sensor for fingerprint authentication in desktop or network security.
- MFS100 is based on optical sensing technology which efficiently recognizes poor quality fingerprints also.
- MFS100 can be used for authentication, identification and verification functions that let your fingerprint act like digital passwords that cannot be lost, forgotten or stolen.
- Hard optical sensor is resistant to scratches, impact, vibration and electrostatic shock.
- Lowest FAR and FRR NIST complied interoperable template format standards (ANSI378 /ISO19794-2). Compatibility with ISO 19794-4 and ISO 19794-2 for fingerprint capture and verification.
- Apart from FBI and STQC, MFS100 is CE, FCC, RoHS, WHQL, CB 60950 approved. Equivalent to FBI PIV certification.
- Supports operating system plaforms like Windows 98 SE, Me, 2000, XP, Windows7, windows vista, windows server(2003/2007/2008) and Linux.
- SDK, Libraries and drivers support across all mentioned platforms. (32 bit/64 bit) Easy integration into product servers and application support

Why the name?

This project was created by researchers at the London School of Hygiene & Tropical Medicine. Our mission is to improve health and health equity in the UK and worldwide; working in partnership to achieve excellence in public and global health research, education and translation of knowledge into policy and practice. LSHTM's main building is situated on Keppel Street in London's Bloomsbury district. The Keppel App and CLI are named after the street where you will find us and not after any (no doubt problematic) historical figures who may have given their name to the street.

Funding & Ethics

This work was funded by the UK Department of Health and Social Care using UK Aid funding managed by the

NIHR (PR-OD-1017-20001). Ethical permission for elements of the work that handled fingerprint templates from living humans was granted by the London School of Hygiene & Tropical Medicine Observational Research Ethics Committee (Ref. 22562).

FUNDED BY
NIHR | National Institute for Health and Care Research
