# Supplementary material for: Biometric linkage of longitudinally collected electronic case report forms and confirmation of subject identity: an open framework for ODK and related tools
Source: Front Digit Health. 2023 Aug 4;5:1072331. doi: 10.3389/fdgth.2023.1072331 (PMC10436742; doi:10.3389/fdgth.2023.1072331)
Supplement: Supplementary file 4 [file Datasheet4.pdf]

How SourceAFIS algorithm works

SourceAFIS » Algorithm

SourceAFIS algorithm is mostly about *understanding* fingerprints. Of course, SourceAFIS is not a human, so how could it understand anything? For algorithms, understanding data means describing it with high-level abstractions. In case of SourceAFIS, these high-level abstractions are *minutiae*, or ridge endings and bifurcations. Minutiae are simply points on the image with associated direction angle.

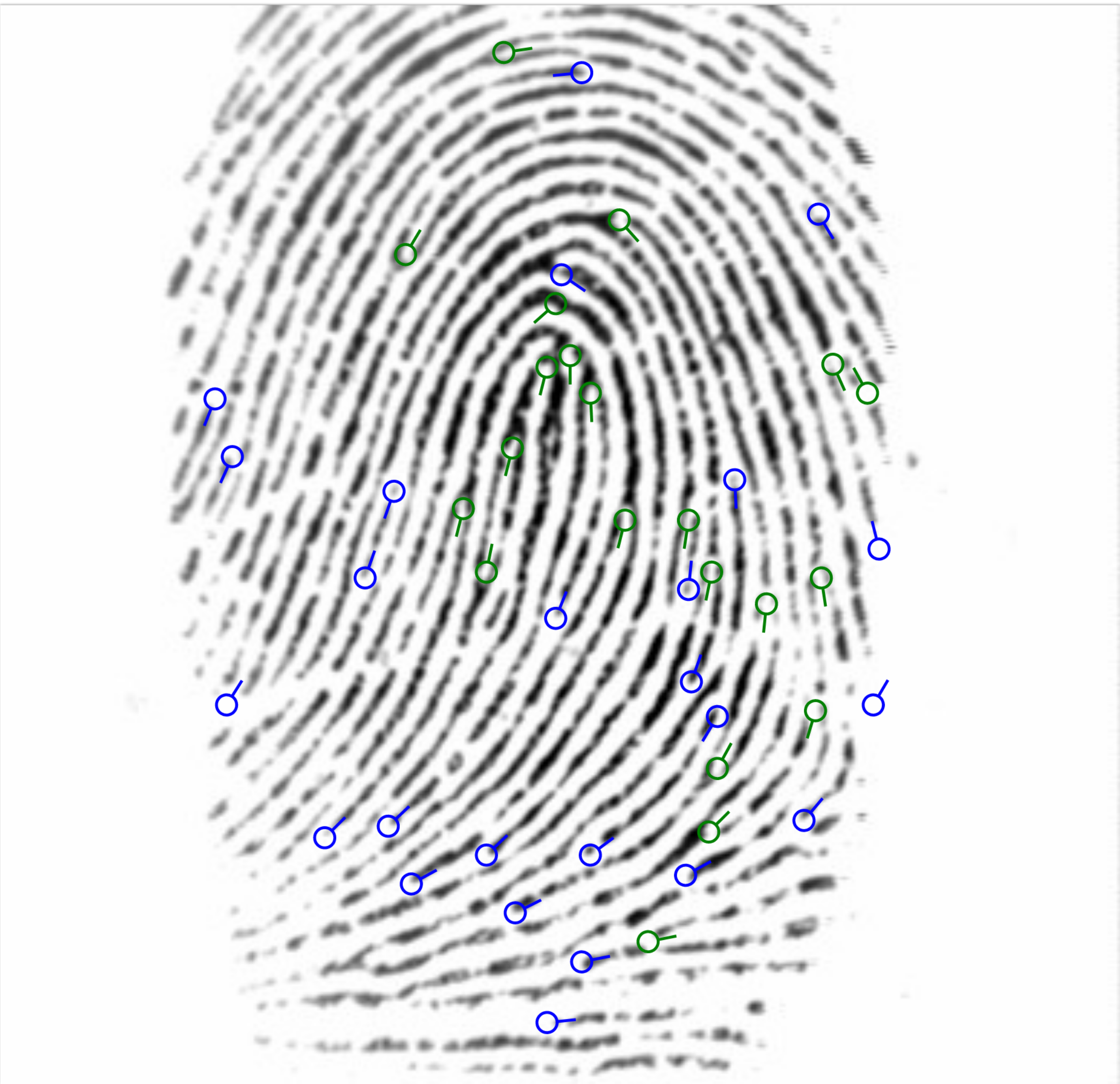

Minutiae found on the fingerprint image.

This is essentially what gets saved in the template. Many small abstractions happen along the way from fingerprint image to the list of minutiae (the template), but we will talk about that some other time.

After minutiae, there is one more abstraction step, which produces *edges*. Edge is a line connecting two minutiae. Edge has length and two angles inherited from its minutiae. Edge angles are expressed as relative to the edge. These three properties of the edge (length and two relative angles) do not change when the edge is moved or rotated and that's exactly what we need for matching.

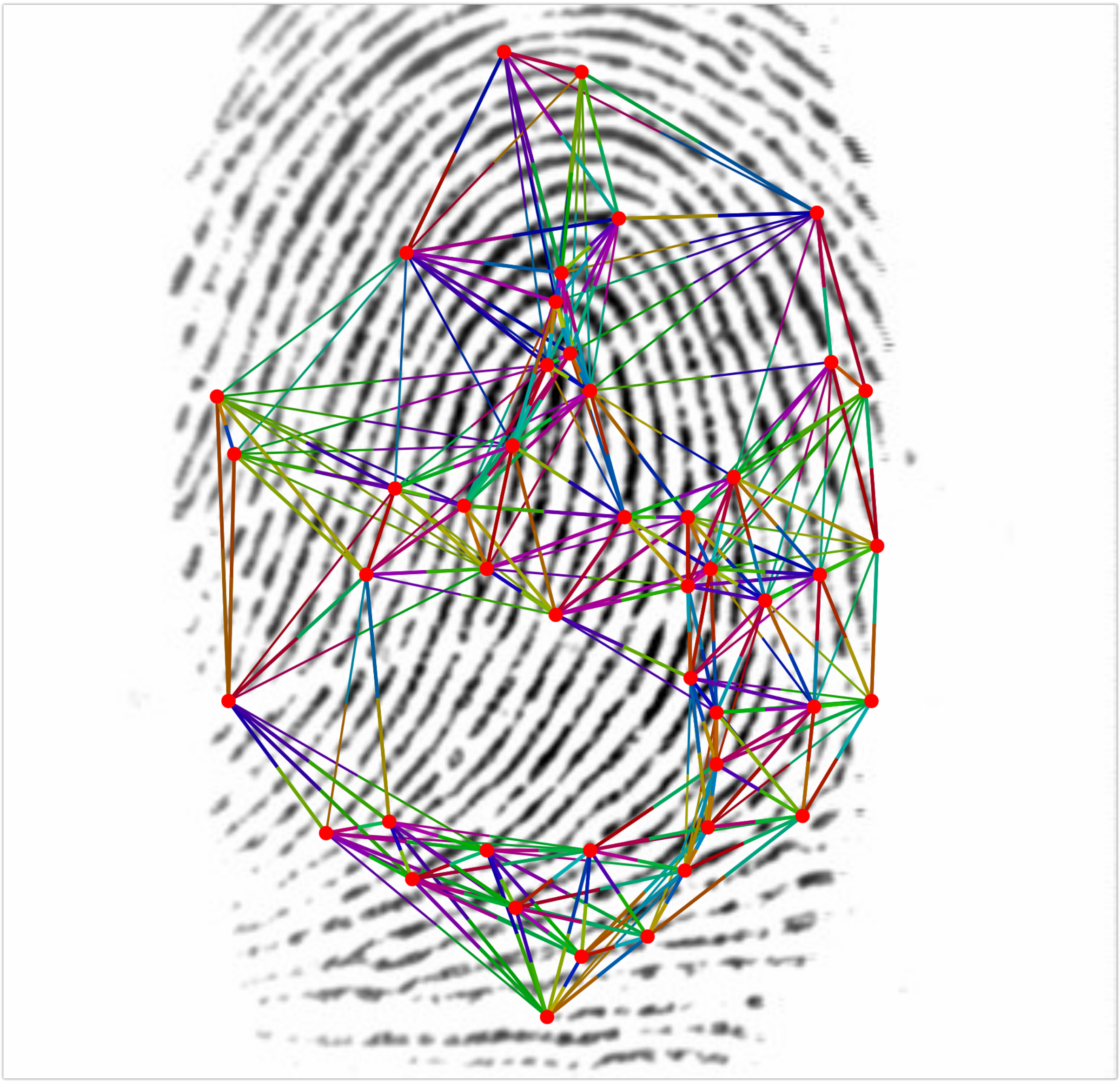

Color is determined by edge length and angles. Similar edges have similar colors.

SourceAFIS then tries to find at least one edge shared by the two fingerprints being matched. This is done very quickly using a nearest neighbor algorithm that has performance comparable to a hash table. That will give us the *root pair*, which is the initial pair of matched minutiae, one from each fingerprint.

Starting from the root pair, SourceAFIS crawls edges outwards and builds a *pairing* consisting of a number of paired minutiae and paired edges.

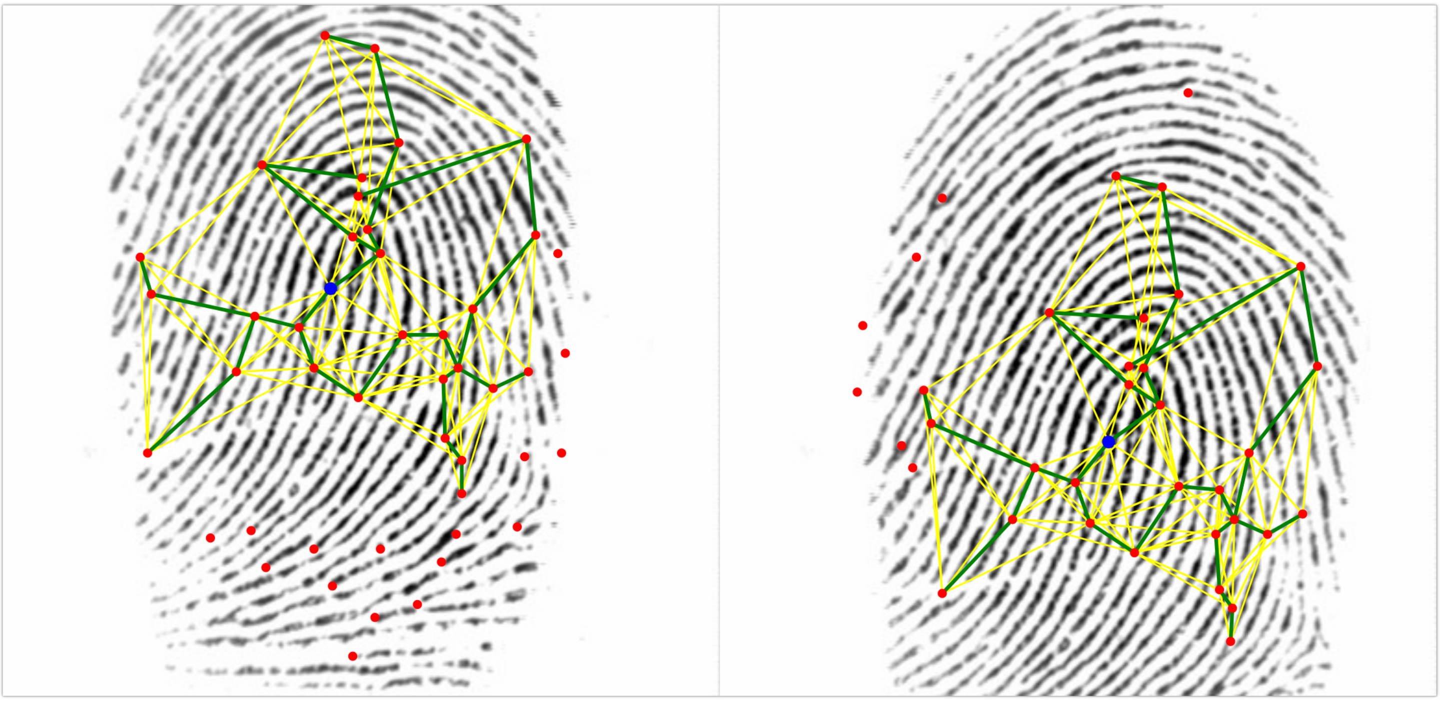

Root minutiae are blue. Pairing tree is green. Graph of supporting edges is yellow.

SourceAFIS now looks carefully at the pairing and decides whether such pairing means a match or whether it's just a coincidence. Of course, everything could be a coincidence, but the difference between weak and strong match is that strong match is very unlikely to be a coincidence.

This is where SourceAFIS runs *scoring*, the last part of the algorithm. The basic idea is that every paired minutia or edge is an event that is unlikely to happen randomly. The more of such unlikely events there are, the less likely the pairing is to be just a coincidence. So the algorithm essentially counts various matched features and also scores them on how closely they match. Final sum of the partial scores is shaped to align to some reasonable scale and returned from the algorithm. Application takes the score and compares it to some *threshold* to decide whether it's a match or not.

If you are still hungry for information, take a look at template format and algorithm transparency. Reading the source code is the definitive way to answer all your remaining questions.

Robert Važan robert.vazan@tutanota.com
