# Supplementary figures and images for: Biometric linkage of longitudinally collected electronic case report forms and confirmation of subject identity: an open framework for ODK and related tools
Source: Front Digit Health. 2023 Aug 4;5:1072331. doi: 10.3389/fdgth.2023.1072331 (PMC10436742; doi:10.3389/fdgth.2023.1072331)

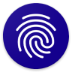

Supplement: Supplementary file 5 [file Datasheet5.zip › Data Sheet 5_v1/ODK_Biometrics-master/Android/app/src/main/res/mipmap-hdpi/ic_launcher.png]

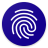

Supplement: Supplementary file 5 [file Datasheet5.zip › Data Sheet 5_v1/ODK_Biometrics-master/Android/app/src/main/res/mipmap-mdpi/ic_launcher.png]

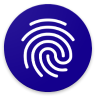

Supplement: Supplementary file 5 [file Datasheet5.zip › Data Sheet 5_v1/ODK_Biometrics-master/Android/app/src/main/res/mipmap-xhdpi/ic_launcher.png]

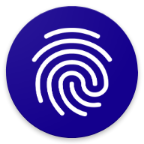

Supplement: Supplementary file 5 [file Datasheet5.zip › Data Sheet 5_v1/ODK_Biometrics-master/Android/app/src/main/res/mipmap-xxhdpi/ic_launcher.png]

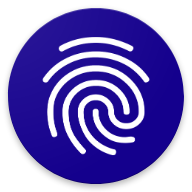

Supplement: Supplementary file 5 [file Datasheet5.zip › Data Sheet 5_v1/ODK_Biometrics-master/Android/app/src/main/res/mipmap-xxxhdpi/ic_launcher.png]

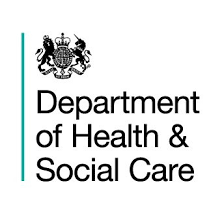

Supplement: Supplementary file 5 [file Datasheet5.zip › Data Sheet 5_v1/ODK_Biometrics-master/imgs/DHSC.png]

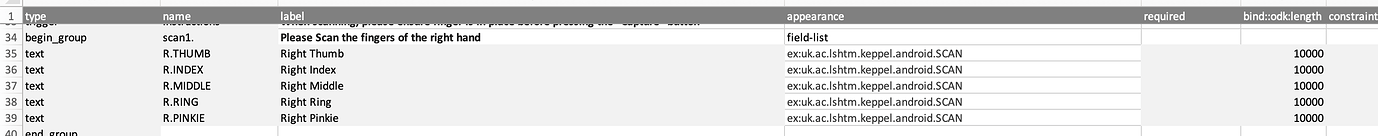

Supplement: Supplementary file 5 [file Datasheet5.zip › Data Sheet 5_v1/ODK_Biometrics-master/imgs/form_five_fingers.png]

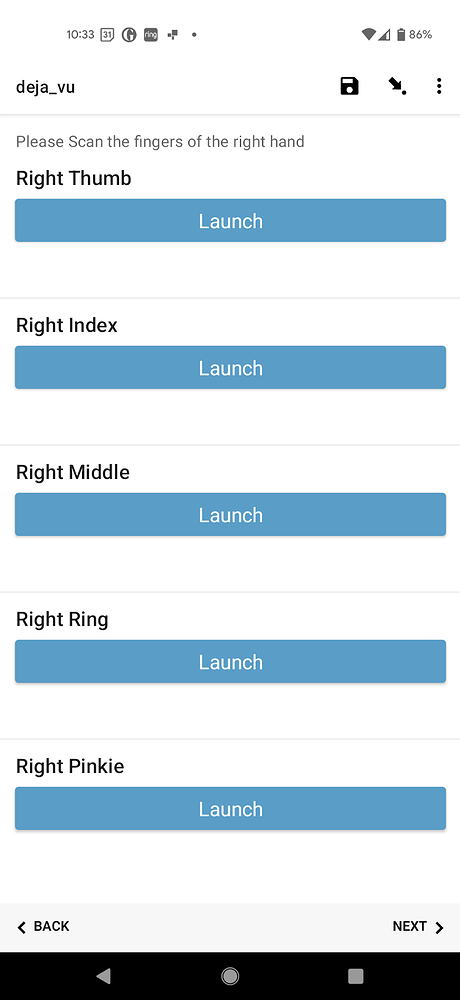

Supplement: Supplementary file 5 [file Datasheet5.zip › Data Sheet 5_v1/ODK_Biometrics-master/imgs/form_five_fingers_odk_collect.png]

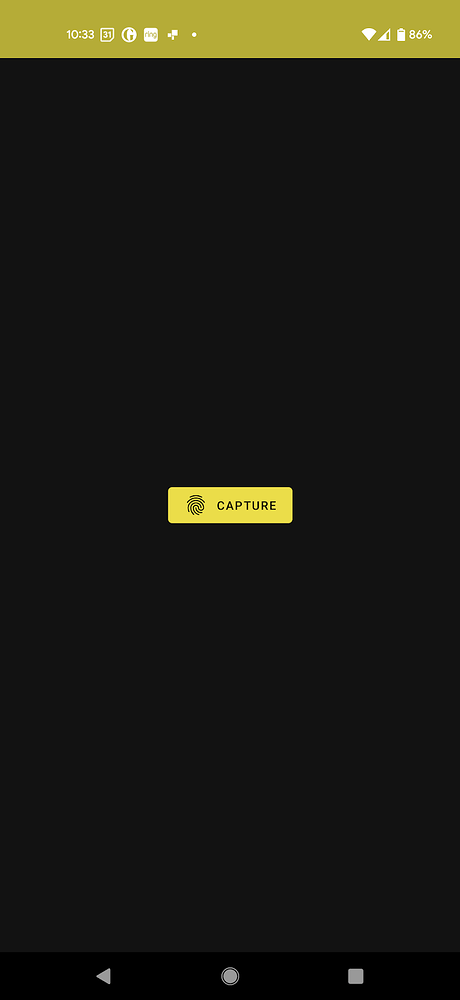

Supplement: Supplementary file 5 [file Datasheet5.zip › Data Sheet 5_v1/ODK_Biometrics-master/imgs/keppel_app_ss_1.png]

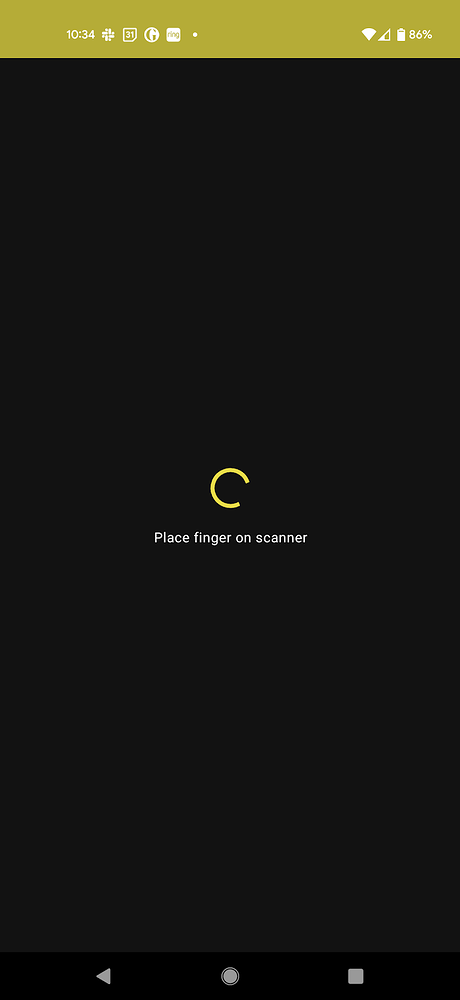

Supplement: Supplementary file 5 [file Datasheet5.zip › Data Sheet 5_v1/ODK_Biometrics-master/imgs/keppel_app_ss_2.png]

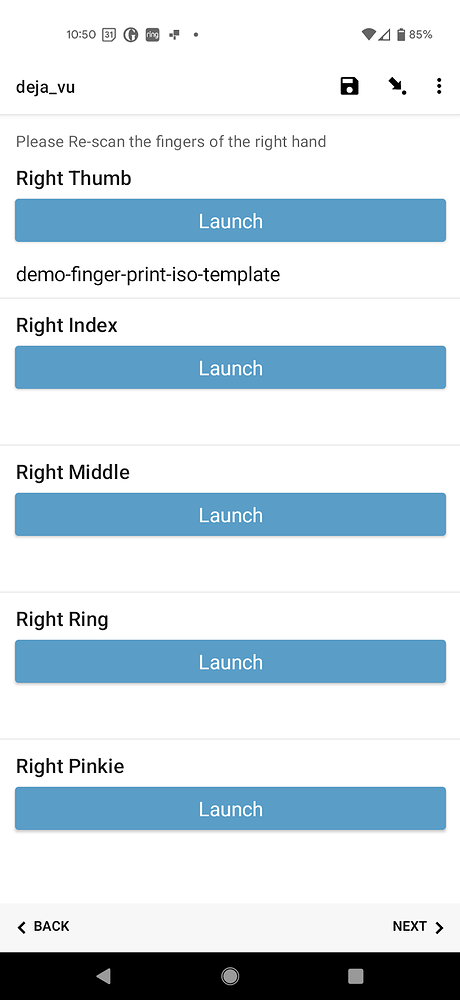

Supplement: Supplementary file 5 [file Datasheet5.zip › Data Sheet 5_v1/ODK_Biometrics-master/imgs/keppel_app_ss_3.png]

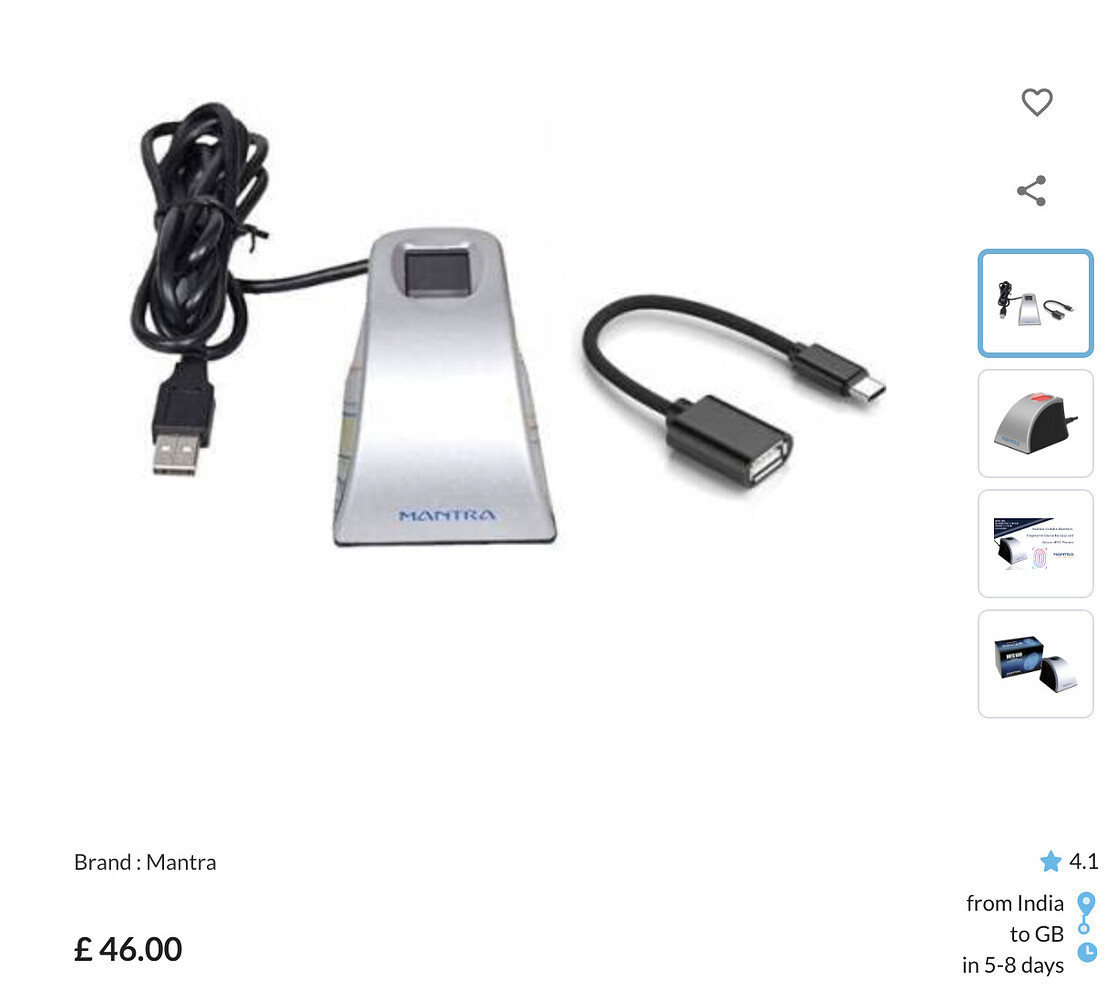

Supplement: Supplementary file 5 [file Datasheet5.zip › Data Sheet 5_v1/ODK_Biometrics-master/imgs/mantra_img.jpeg]

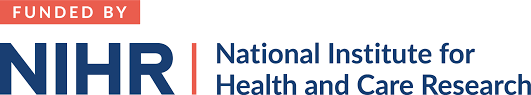

Supplement: Supplementary file 5 [file Datasheet5.zip › Data Sheet 5_v1/ODK_Biometrics-master/imgs/NIHR.png]
